# Acadesine Kills Chronic Myelogenous Leukemia (CML) Cells through PKC-Dependent Induction of Autophagic Cell Death

**DOI:** 10.1371/journal.pone.0007889

**Published:** 2009-11-18

**Authors:** Guillaume Robert, Issam Ben Sahra, Alexandre Puissant, Pascal Colosetti, Nathalie Belhacene, Pierre Gounon, Paul Hofman, Fréderic Bost, Jill-Patrice Cassuto, Patrick Auberger

**Affiliations:** 1 INSERM UMR 895, Team 2: Cell Death Differentiation and Cancer, Nice, France; 2 INSERM UMR 895, Team 7, Nice, France; 3 Equipe labellisée par la Ligue Nationale contre le Cancer, Paris, France; 4 Centre Commun de Microscopie Appliquée, Faculté des Sciences, Parc Valrose, Nice, France; 5 INSERM ERI-21/EA4319, Faculty of Medicine and Laboratory of Clinical and Experimental Pathology, CHU Nice, BP 69, Nice, France; 6 Centre Hospitalier Universitaire de Nice, Hopital de l'Archet, Service d'Hématologie Clinique, Nice, France; 7 Université de Nice Sophia-Antipolis, UFR Médecine, Nice, France; Technical University Munich, Germany

## Abstract

CML is an hematopoietic stem cell disease characterized by the t(9;22) (q34;q11) translocation encoding the oncoprotein p210BCR-ABL. The effect of acadesine (AICAR, 5-Aminoimidazole-4-carboxamide-1-β-*D*-ribofuranoside) a compound with known antileukemic effect on B cell chronic lymphoblastic leukemia (B-CLL) was investigated in different CML cell lines. Acadesine triggered loss of cell metabolism in K562, LAMA-84 and JURL-MK1 and was also effective in killing imatinib-resistant K562 cells and Ba/F3 cells carrying the T315I-BCR-ABL mutation. The anti-leukemic effect of acadesine did not involve apoptosis but required rather induction of autophagic cell death. AMPK knock-down by Sh-RNA failed to prevent the effect of acadesine, indicating an AMPK-independent mechanism. The effect of acadesine was abrogated by GF109203X and Ro-32-0432, both inhibitor of classical and new PKCs and accordingly, acadesine triggered relocation and activation of several PKC isoforms in K562 cells. In addition, this compound exhibited a potent anti-leukemic effect in clonogenic assays of CML cells in methyl cellulose and in a xenograft model of K562 cells in nude mice. In conclusion, our work identifies an original and unexpected mechanism by which acadesine triggers autophagic cell death through PKC activation. Therefore, in addition to its promising effects in B-CLL, acadesine might also be beneficial for Imatinib-resistant CML patients.

## Introduction

CML is a myeloproliferative syndrome linked to a hematopoietic stem cell disorder leading to increased production of granulocytes at all stages of differentiation [1]. CML patients carry the t(9;22) (q34;q11) translocation [1], which is responsible for the expression of p210 BCR-ABL, a constitutively active tyrosine kinase [2]. The role of BCR-ABL in the pathogenesis of CML is well documented [3], [4]. Of note, BCR-ABL mediates several survival pathways including STAT5/Bcl-xL, Ras/Raf/MEK/Erk-1/2, PI3K/Akt and NF-kB, that collectively confer proliferative advantages and resistance to apoptosis [5], [6], [7], [8]. Imatinib (Gleevec) which targets the ATP-binding site of different tyrosine kinases including BCR-ABL [9], [10], selectively induces growth arrest and apoptosis of BCR-ABL positive leukemia cells with minimal effect on normal hematopoietic progenitors [11], [12], [13].

AMP-activated protein kinase (AMPK) is a serine/threonine protein kinase that regulates the intracellular AMP/ATP ratio and participates in the regulation of glucose and lipid metabolism [14], [15]. Although Acadesine is commonly used as an AMPK activator, there are compelling evidence in the literature that the acadesine anti-tumoral effects could be not mediated by the AMPK pathway [16], [17], [18]. Nevertheless, at present, the exact nature of the AMPK-independent effects of acadesine in leukemic cells is not understood.

In this study, we investigated the AMPK pathway as a possible target for therapeutic intervention in CML patients. We found that acadesine exerts a potent antileukemic effect in CML cell lines either sensitive or resistant to Imatinib. Unexpectedly, the acadesine anti-proliferative effect involved neither caspase activation nor apoptosis. Moreover, whereas acadesine consistently activated the AMPK pathway in CML cell lines, its anti-proliferative effect was found to be independent on AMPK, rather, activation of PKC was found to be required. Importantly, the anti-leukemic effect of acadesine involved autophagy. To our knowledge, our study provides the first evidence that acadesine exerts its anti-leukemic through induction of autophagic cell death. Finally, acadesine also inhibited tumor formation in a xenograft model of CML in nude mice. Hence, acadesine may find therapeutical application in Imatinib-resistant CML patients.

## Materials and Methods

### Reagents and Antibodies

RPMI 1640 medium and fetal calf serum (FCS) were purchased from Gibco BRL (Paisley, UK). Sodium fluoride, orthovanadate, phenylmethylsulfonyl fluoride, aprotinin and leupeptin were purchased from Sigma (Saint-Louis, MO, USA). Ac-DEVD-AMC, Z-RR-AMC, Ac-DEVD-CHO were purchased from Alexis Biochemicals (Lausan, Switzerland). GF109293X (GFX) and Ro 32-0432 were from Tocris (Bristol, UK). Anti-SQSTM1, anti-Actin, anti-Hsp90, anti-Hsp60 and anti-Abl antibodies were from Santa Cruz Biotechnology (Tebu-Bio, Le Perray en Yvelines, France). Anti-LC3B, anti-AMPK, anti-phospho-AMPK (Thr172), anti-phospho-S6 Ribosomal Protein (Ser235/236), anti-S6 Ribosomal Protein, anti-phospho-Abl (Tyr245) and anti-phospho-Crkl (Tyr207) were purchased from cell signalling technology (Danvers, MA, USA). HRP conjugated anti-mouse, anti-rabbit and anti-goat antibodies from Dakopatts (Glostrup, Denmark).

### Cell Lines

The human CML K562, LAMA-84 and JURL-MK1 cell lines were provided by ATCC and were grown at 37°C under 10% CO_2_ in RPMI 1640 medium (Gibco BRL, Paisley, UK) supplemented with 5% FCS (Gibco BRL, Paisley, UK) completed with 50 units/ml penicillin, 50 µg/ml streptomycin and 1 mM sodium pyruvate. Imatinib resistant K562 cells was described earlier [19]. The Ba/F3 p210BCR/ABL WT and T31I cells were kindly provided by Pr. FX Mahon and Pr. JV Melo.

### Caspase 3 Activity Measurements

Caspase 3 assay has been described earlier [20], [21].

### Measurement of Cell Metabolism (XTT)

Cells (15×10^3^ cells/100 µl) were incubated with the effectors for the times indicated. 50 µl of XTT reagent (sodium 3′-[1-(phenylaminocarbonyl)-3,4-tetrazolium]-bis(4-methoxy-6-nitro) benzene sulfonic acid hydrate) was added to each well. Absorbance of the formazan dye produced by metabolically active cells was measured at 490 nm as described earlier [19]. Each assay was performed in quadruplicate.

### Western Blot

Western blot analysis have been described in details previously [19].

### Electronic Microscopy

K562 cell pellets were collected, fixed with 1.6% glutaraldehyde, post-fixed in 1% OsO_4_, dehydrated in alcohol series, and embedded in epoxy resin. Thin sections were contrasted with uranyl acetate and lead citrate. Preparations were observed either with a Philips CM12 electron microscope operating at 80 kV (FEI, Eindhoven, The Netherlands) or with a Jeol 1400 (Tokyo, Japan) mounted with CCD cameras (Morada, Olympus SIS, Germany). Samples were analysed with Jeol 1200 XII Philipps electron microscope [6].

### Preparation of Cytoplasmic and Microsomal Fractions and Detection of PKC Activation

After stimulation with PMA or Acadesine, cells were washed with wash buffer (proteoExtract® Subcellular Proteome Extraction KIT, Calbiochem, La Jolla, CA, USA) and pelleted by centrifugation for 10 min at 300 g. Extraction Buffer I was added and cells were incubated for 10 min at 4°C under gentle agitation and centrifugated for 10 min at 800 g. The supernatant contains the cytoplasmic fraction. Extraction Buffer II was added to the pellet and cells were incubated for 30 min at 4°C under gentle agitation. Finally they were centrifugated for 10 min at 5500 g. The supernatant contains the microsomal fraction was collected. Cytosolic and Lysosomal fractions were separated by SDS-Page and transferred onto PVDF membranes which were incubated with specific anti-PKC α, β, γ, θ antibodies from BD transduction laboratories (Franklin Lakes, NJ, USA) [7].

### CB Activity Measurement

After stimulation, cells were lysed for 30 min at 4°C in lysis buffer (0.4 M Na Phosphate PH 6, 150 mM NaCl, 4 mM EDTA, 1 mM PMSF, 10 µg/ml aprotinin and 1% Triton X-100) and lysates were cleared at 10000 g for 15 min at 4°C. Each assay was performed with 50 µg of protein prepared from control or stimulated cells. Cellular extracts were incubated with 60 µM of z-RR-AMC as substrate for various times at 37°C. CB activity was measured by following emission at 460 nm (excitation at 390 nm) as described earlier [19].

### Primary Cells Isolation

Blood samples were collected from patients newly diagnosed for CML as part of an institutionally approved cellular sample collection protocol. Informed consent has been obtained according to institutional guidelines. Mononuclear cells were isolated from blood samples by density centrifugation (Ficoll-Paque™ Plus), washed with PBS, 5% SVF, 2 mM EDTA and resuspended in cell culture medium (IMDM, 10% fetal bovine serum) and incubated overnight at 37°C in a 5% CO_2_ incubator before CD34+ cells isolation. CML cells were labelled with CD34 microbeads isolated by magnetic positive selection (StemSep™ Human CD34 Selection Kit; StemCell, Vancouver, BC, Canada). Purity was estimated to At least 90% by FACS analysis. Experiments were performed using a StemSpan^R^ SFEM medium (StemCell, Vancouver, BC, Canada) supplemented with 100 ng/ml human recombinant SCF, FLT3-L and 20 ng/ml human recombinant IL-3, IL-6 and G-CSF (Peprotech, Rocky Hill, NJ, USA).

### Colony Formation Assay

Acadesine was added to K562 cell lines or primary cells (10^3^ CD34+ cells/ml) growing in semisolid methyl cellulose medium. MethoCult H4100 or H4236 were used for cell lines and primary CD34+ cells respectively (StemCell Technologies Inc., Vancouver, Canada). Colonies were detected after 10 days of culture by adding 1 mg/ml of 3-(4,5-dimethylthiazol-2-yl)-2,5-diphenyltetrazolium bromide (MTT) reagent and were scored by Image J quantification software (U.S. National Institutes of Health, Bethesda, MD, USA).

### Confocal Microscopy Experiments

K562 cells were treated with MitoTracker® Red and Lysotracker® Green for 30 min at 37°C.Then cells were washed with PBS and cytospun on a slide with Cytofunnel® (Thermo Fisher Scientific Inc., MA, USA). Cells were fixed with paraformaldehyde 3% for 10 min and after fixation and rinsed several times with PBS. Finally slides were analyzed with Confocal microscope ZEISS LSM510 META.

### Sh-RNA Assays

K562 cells were transfected by electroporation. Cells were centrifuged at 400 g for 5 min and resuspended in 100 µl of buffer V containing 2 µg of empty vector or plasmid expression vector coding for sh-RNA targeting AMPK (Sigma, St Louis, MO, USA). Cells were electroporated using the T-16 program of the Amaxa nucleofector (Amaxa, Koln, Germany). 48 h after transfection, cells were treated with 1 mM acadesine. 48 h latter, cell metabolism assays were realized and Western Blots were performed to check extinction of AMPK expression.

### si-RNA Assays

siRNA transfections were performed using Lipofectamine RNAiMAX (Invitrogen). K562 cells were centrifuged at 400 g for 5 min and resuspended in RMPI with 5% FCS completed with 1 mM sodium pyruvate. Then, cells were transfected with 50 nM of si-AMPKα1 and si-AMPKα2 or si-Control. After 48 h, cells were stimulated with 1 mM of acadesine or 1 µM of imatinib. Two day later, cell metabolism assays were carried out and Western Blots were performed to check extinction of AMPK expression.

### Tumor Regression Experiments in Nude Mice

Female Nude NMRI Mice (Janvier, Le Genest Saint Ile, France) were randomized into two experimental groups, each containing 15 animals. Animals in both groups received a 100 µl injection of 5.10^6^ K562 leukemia cells on both flanks. When tumors reached 150–200 mm^3^, animals were injected intraperitoneally with NaCl 0.9% or acadesine at dose level of 50 mg/kg body weight. The volume of tumors were measured every 5 days Tumor volume was calculated according to the mathematical formula: V = (0.4)*L*(W)^2^, (L: Length; W: Width).

### May-Grünwald Giemsa Staining

K562 cells were processed as described previously [6].

### Measurement of Apoptosis

After Imatinib or acadesine stimulation, K562 and Ima-R cells were stained according to manufacturer's recommended protocol for Annexin-V-FLUOS Staining Kit (Roche Diagnostics, Penzberg, Germany).Then, staining cells were analyzed with cytometer.

## Results

### Acadesine-Mediated Inhibition of Cell Viability Does Not Involve Apoptosis

**Figure 1:**
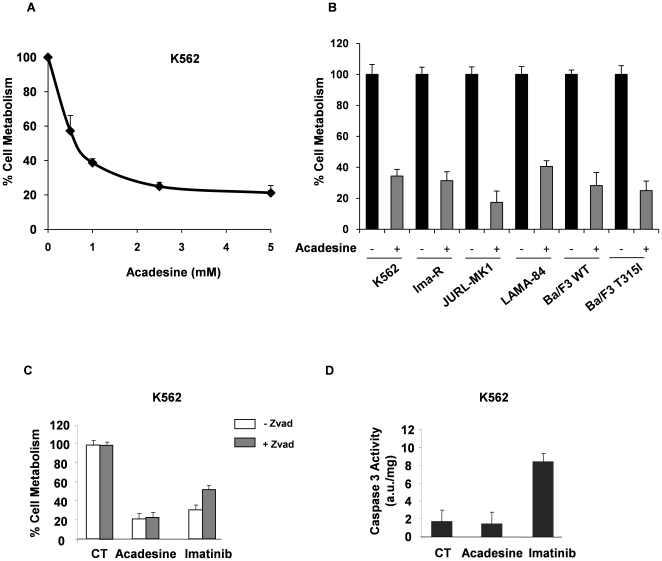
Acadesine Induces loss of cell viability in an apoptosis independent manner. (A) K562 cells were incubated for 48 h at 37°C with increasing concentrations of acadesine. Cell metabolism was measured by the XTT assay as described in Materiel and Methods section. Results are mean±SD of 4 different determinations made in quadruplicate. Error bars = 95% confidence intervals. (B) CML cells or Ba/F3 cells (WT or T315I) were treated with 1 mM acadesine and cell viability was determined using the XTT assay. (C) K562 cells were incubated for 48 h at 37°C with 1 mM acadesine or 1 µM Imatinib in the presence or absence of 50 µM zVAD-fmk (ZVAD). Cell metabolism was determined as described above. (D) K562 cells were incubated as described in (C). Cells were harvested, washed, and lysed in caspase buffer. Caspase-3 activity was evaluated in quadruplicate using Ac-DEVD-AMC as substrate. To allow specific assessment of caspase activity, hydrolysis was followed as a function of time in the presence or the absence of 10 µM Ac-DEVD-CHO. Results, expressed as arbitrary units (a.u.) per mg of proteins are the means±SD of 4 independent experiments performed in quadruplicate. Error bars = 95% confidence intervals.

To investigate the effect of acadesine on cell metabolism, we stimulated different CML cell lines for 48 h with various concentrations of this compound. Acadesine induced a dose-dependent decrease of cell metabolism with a maximal effect around 1 mM in all the CML cell lines tested (Figures 1A, B and Figure S1 A to C). Therefore, all the forthcoming experiments were performed with this concentration of acadesine. Importantly, acadesine also inhibited cell metabolism in imatinib-resistant K562 cells and in Ba/F3 cells carrying the BCR-ABL-T315I mutation (Figures 1B and Figure S1D). Next, we investigated whether acadesine exerted its anti-leukemic effect through induction of apoptosis. As expected, z-VAD-fmk inhibited by 30–40% Imatinib-mediated loss of cell metabolism in K562 cells at 48 h [22], whereas it failed to reduce the effect of acadesine (Figure 1C). Accordingly, and in contrast to Imatinib, acadesine neither activated caspase 3 (Figure 1D) nor it induced phosphatidyethanolamine externalisation in K562 and other CML cells (Figure S1 E and F). Therefore, we conclude that apoptosis is not required for acadesine-mediated inhibition of cell metabolism in several well characterized CML cell lines.

### Morphological Analysis of Acadesine-Treated K562 Cells

**Figure 2:**
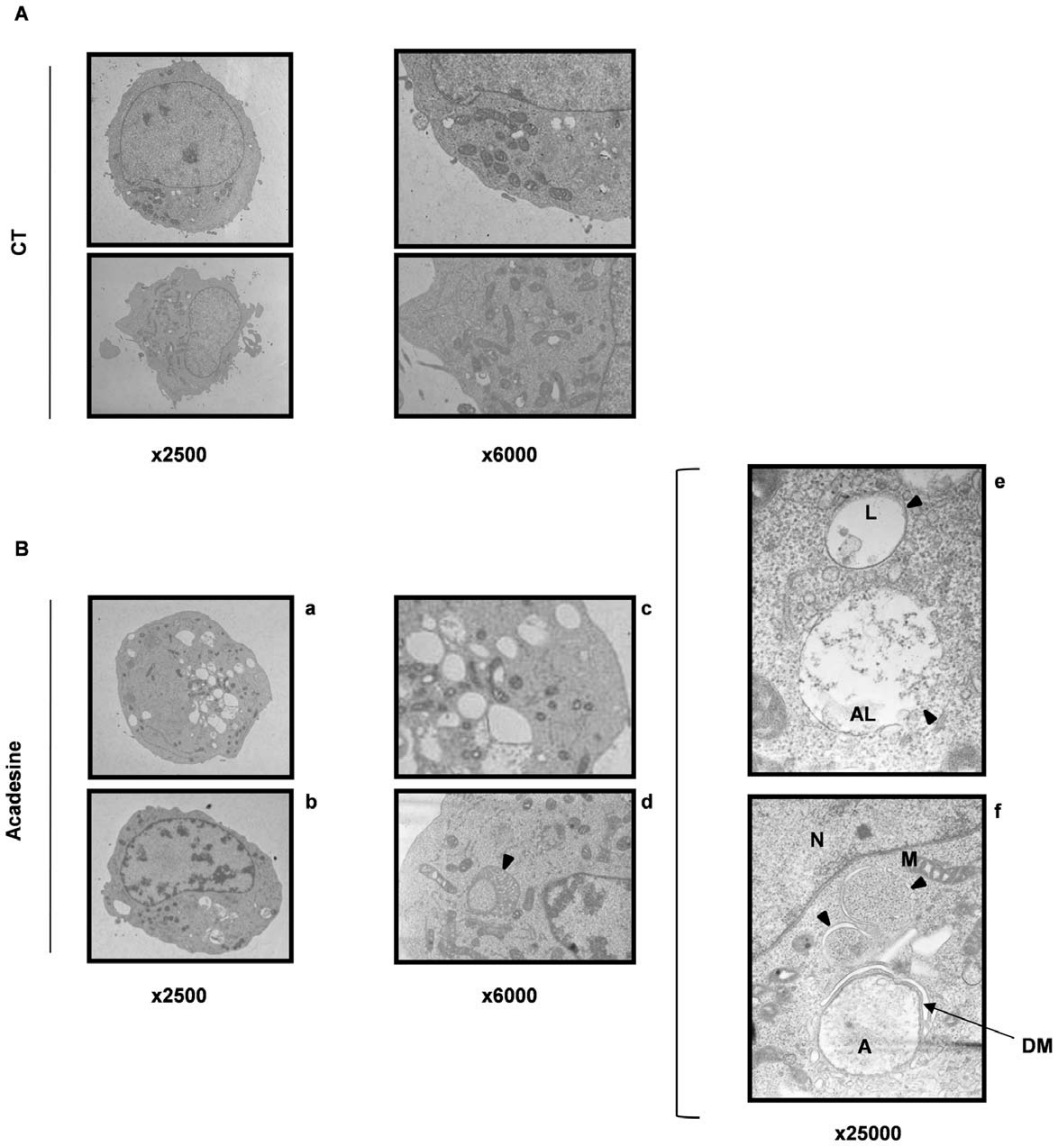
Acadesine induces vacuole formation and degradation of cytoplasmic material. Electron microscopy images showing ultrastructural features of a representative control cell (A) and morphological features of autophagy in K562 treated with 1 mM acadesine for 48 h (B). Cells were observed at different magnification (x2500, x6000 and x25000). L =  Lysosome, A =  Autophagosome, N =  Nuclei, M =  Mitochondria, DM =  Double Membrane Vesicle.

**Figure 3:**
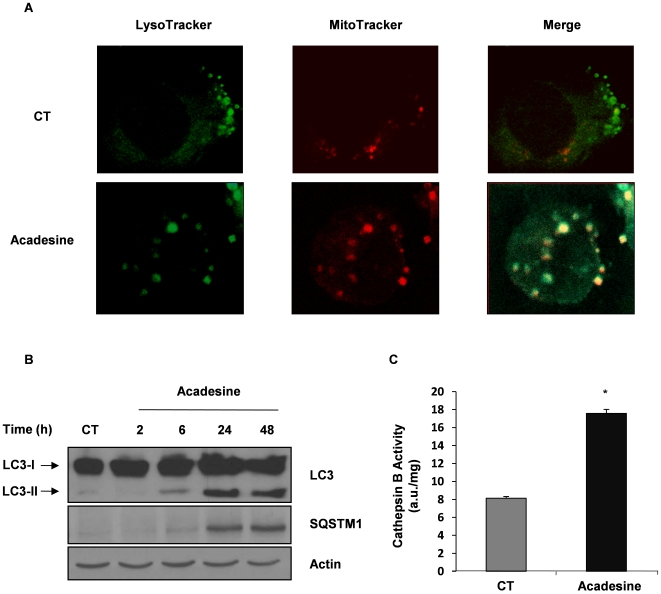
Acadesine induces hallmarks of autophagy in K562 cells. (A) K562 cells were incubated at 37°C with 1 mM acadesine. After 48 h cells, were incubated for 30 min with the Mitotracker-Red 580 and the Lysotracker-Green DND-189. They were cytospun on a slide fixed with 3% paraformaldehyde, mounted on glass slides and analysed by confocal microscopy. (B) K562 cells were incubated at 37°C with 1 mM acadesine for the indicated times. Whole-cell lysates were prepared, and expression of SQSTM1 and LC3 was visualized by western blotting. Actin was used as loading control. (C) K562 cells were incubated with 1 mM acadesine for 48 h at 37°C and cathepsin B activity was evaluated in the presence or the absence of CA-074Me as described in Materials and Methods section. Results, expressed as arbitrary units (a.u.) per mg of proteins are the means±SD of 4 independent experiments performed in quadruplicate. Error bars = 95% confidence intervals.

To gain insight into the anti-leukemic effect of acadesine, we performed May-Grunwald Gemsa staining of K562 cells. Cells treated for 48 h with acadesine revealed marked morphologic modifications, including increase in both cell and nucleus sizes (Figure S2A). Moreover, accumulation of small to large size vacuoles was consistently detected in acadesine-treated cells (Figures 2A and B). These vacuoles were detected as soon as 24 h of acadesine treatment with a maximal accumulation at 72 h (Figure S2B). Such vesicle formation has been recently documented in K562 cells undergoing autophagy [23]. To characterize further these vacuoles, we next carried out electron microscopy experiments. Acadesine increased drastically the number and the size of the vacuoles present in K562 cells (Figure 2A and B). They were concentrated in the viscinity of mitochondria and sometimes, mitochondria were found to be included in vacuoles (Figure 2B). Larger magnification showed typical lysosomes (Figure 2B) and autophagosomes (Figure 2B, arrow). To demonstrate further that these vacuoles do represent autophagosomes and whether mitochondria inclusion in these putative vesicles reflects an autophagic process, we incubated K562 cells with acadesine for 48h before addition of the lysosensor-GREEN DN-189 or the mitotracker red 580 and performed confocal microscopy experiments. In untreated K562 cells, numerous lysozomes and some mitochondria were seen (Figure 3A). Importantly, while the two dyes clearly segregated in untreated cells, they colocalized frequently in acadesine-treated cells. These findings suggest that acadesine induces mitophagy in CML cell lines. Hence, we analyzed whether acadesine might induce characteristic hallmarks of autophagy. During autophagy, LC3-I is cleaved to LC3-II and subsequently modified through the Atg4-dependent insertion of a phosphoethanolamine moiety before its insertion into the membrane of the forming phagophore, a double membrane required for the recycling of protein aggregates and organelles [24]. Increased LC3 expression was detected as soon as 6 h after acadesine treatment, with a maximal accumulation at 24 h (Figure 3B). In the meantime, acadesine also enhanced the expression level of P62/SQSTM1 a scaffold protein involved in the elimination of protein microaggregates through autophagy. These results were confirmed using the same CML cell lines used previously (Figure S3 A–C). The observation that acadesine affected the lysosomal compartment was further substantiated by the increase in Cathepsin B (CB) activity mediated by this compound at 48 h in K562 cells (Figure 3C). Importantly, increased CB activity has been associated with the late steps of autophagy [25].

### The Anti-Leukemic Effect of Acadesine Is Independent on AMPK Activation

**Figure 4:**
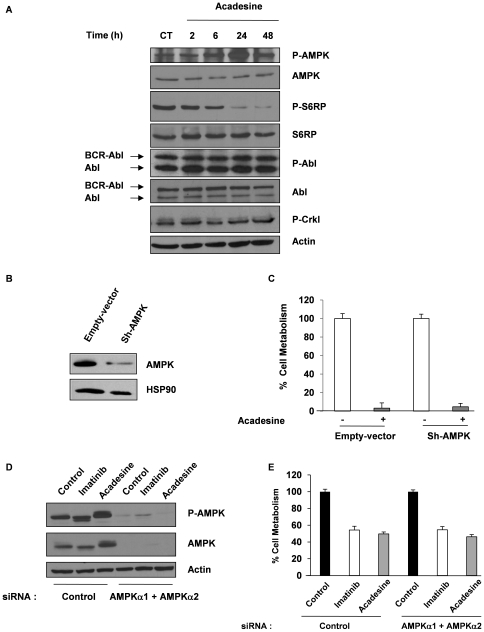
Inhibition of the acadesine-induced AMPK pathway fails to rescue cell viability. (A) K562 cells were incubated with 1 mM Acadesine for the times indicated. Cells were harvested, washed and lysed in lysis buffer. Protein samples were separated by electrophoresis and expression of Phospho-AMPK, AMPK, Phospho-S6 Ribosomal Protein, S6 Ribosomal Protein, phospho-Abl, Abl and phospho-Crkl was visualized by western blotting. (B) K562 cells were transfected by electoporation with either an empty vector or a plasmid expression vector coding for a sh-RNA targeting AMPK. 48 h later, cells were treated or not with 1 mM acadesine. Protein samples were prepared and separated by SDS-Page and the proteins of interest were visualized by western blotting. (C) K562 cells were transfected as in (B) and cell metabolism was measured by the XTT assay as in Figure 1A. In panels A and B, Actin and HSP90 were used as loading control. (D) K562 cells were transfected with control siRNA or the combination of α1 and α2 AMPK siRNA. 48 h later, cells were treated with 1 µM imatinib or 1 mM acadesine for 48 h. Finally, cells were lysed and lysates were analyzed for AMPK extinction. Actin was used as a loading control. (E) Cells were transfected as described above, and cell metabolism was measured by the XTT assay as in Figure 1A.

There is accumulating evidence in non-hematopoietic tumors that the anti-cancerous effect of AMPK activators, including acadesine, may be AMPK independent [26]. To determine whether acadesine does activate AMPK in CML cells, we analyzed the phosphorylation status of this kinase in K562 cells using an anti-phospho-AMPK specific antibody. Acadesine increased AMPK phosphorylation as soon as 6 h, with a maximal effect at 24 h in different CML cell lines (Figure 4A). It is well established that activation of AMPK is associated with inhibition of the m-TOR pathway leading to ribosomal protein S6 dephosphorylation [27]. Accordingly, acadesine inhibited S6 phosphorylation, in agreement with the observed time-course of AMPK activation. Of note, this compound failed to modulate BCR-ABL and Crkl phosphorylation at the same time, indicating that the acadesine anti-leukemic effect is independent on BCR-ABL. To definitely turn-down the possibility that AMPK could mediate the anti-leukemic effect of acadesine, we knocked-down AMPK protein expression by sh-RNA. Although an 80% decrease in AMPK expression was achieved in K562 cells, 48 h after transfection of the most potent AMPK sh-RNA, this had no effect on acdesine-mediated loss of cell metabolism in K562 cells (Figure 4C). Identical results were obtained using a couple of Si-RNA that inhibits both the α1 and α2 catalytic subunits of AMPK (Figure 4D). Here again, inhibition of AMPK expression failed to increase cell viability in the presence of either acadesine or imatinib, demonstrating that AMPK is not responsible for the loss of cell metabolism in drug-treated cells (Figure 4E and not shown). Hence, the anti-leukemic effect of acadesine in CML cells is not mediated by AMPK.

### PKC Activation Mediates the Inhibitory Effect of Acadesine on Cell Viability

**Figure 5:**
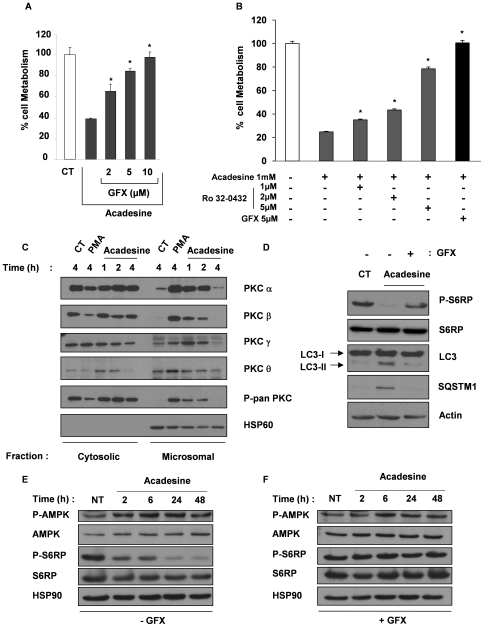
Alteration of leukemic cell viability by acadesine is mediated via PKC activation. (A) K562 cells were incubated with 1 mM acadesine in the presence of different concentrations of GFX. 48 h later, cell metabolism was measured by the XTT assay. (B) K562 cells were incubated with 1 mM acadesine in the presence of increasing concentrations of Ro-32-0432 or 5 µM of GFX. 48 h later, cell metabolism was measured by the XTT assay. (C) K562 cells were incubated with 1 mM acadesine or 20 ng/ml PMA as a positive control of PKC activation. At the times indicated, cells were harvested, washed and subcellular fractions were prepared. Protein samples were separated by electrophoresis and expression of PKCα, β, γ , θ was visualized by western blotting. As expected, HSP60 was found only in the microsomal fraction. (C) Cells were incubated with 1 mM acadesine and treated with or without GFX (5 µM). 48 h later, whole-cell lysates were prepared, and expression of SQSTM1 and LC3 were visualized by western blotting. Actin was used as loading control. (D) Electron microscopy images showing ultrastructural features of representative cells in each condition.

The molecular mechanisms underlying the anti-leukemic effect of acadesine are not known. To decipher further the mechanism of action of this compound in CML cells, we analyzed the effect of a panel of pharmacological agents known to inhibit the main cellular signaling pathways including p38MAPK, JNK, ERK1/2, PI3K, mTOR, PKA, PKC and the caspase cascade. Among them, only GF109293X (GFX), an inhibitor of both classical and new PKC isoforms prevented the inhibitory effect of acadesine on K562 cell viability (Figure 5A). To exclude a non-specific effect of GFX we also used another inhibitor of PKC, namely Ro-32-0432. This confirmed the role of PKC in mediating the acadesine effect (Figure 5B). PKC activation requires relocation from the cytoplasmic to the microsomal fraction [28], [29]. We used this later feature to analyse the effect of acadesine on the expression of the four main PKC isoforms found in K562 cells [7], [30], namely, PKCα, β, γ and θ. 4 h following PMA stimulation, PKCα and β disappeared from the cytoplasmic fraction, concomitantly to their redistribution into the microsomal fraction (Figure 5C). Of note, acadesine also induced, albeit to a lesser extent than PMA, the relocation of PKCα, β andI γ isoforms. The PKC isoforms present in the microsomal fractions were phosphorylated, reflecting their active status. Activation of PKCs was detected as soon as 1 h following acadesine addition, a time point that precedes the onset of autophagy. Importantly, GFX efficiently inhibited acadesine-mediated S6 phosphorylation, LC3 accumulation and SQSTM1 expression illustrating the fact that PKC activation is responsible for induction of autophagy by acadesine in K562 cells (Figure 5D). Involvement of PKC activation in acadesine-mediated autophagy was confirmed using electron microscopy, by the inhibitory effect of GFX on autophagosome and autophagolysosome formation (Figure S4). In an attempt to identify which PKC was involved in the effect of acadesine we knock-downed α, β and γ. Unfortunately, while specific si-RNA inhibited significantly the expression of these isoforms, diminution of any of these isoforms alone was capable to prevent acadesine effect's on cell viability (not shown), suggesting that the combination of several isoforms is necessary to transduce the effect of acadesine.

### Acadesine Inhibits Tumor Formation in a Mouse Xenograft Model of K562 Cells

**Figure 6:**
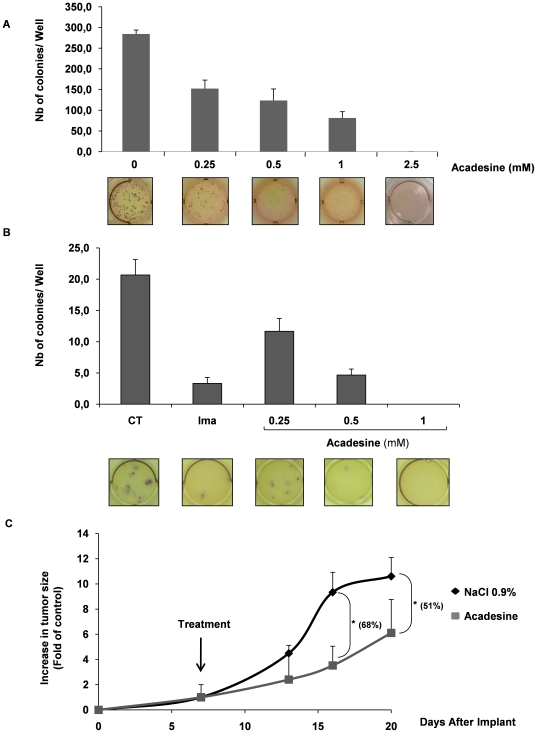
Acadesine inhibits tumor growth in nude mice. (A) Acadesine in the 0.25-2.5 mM range was added to K562 CML cell lines growing in semi-solid methyl cellulose medium (0.5×10^3^ cells/ml). Colonies were detected after 10 days of culture by adding 1 mg/ml of the MTT reagent and were scored by Image J quantification software. Results are expressed as the number of colony forming cells by well after drug treatment in comparison with the untreated control cells (CT). Results are means±SD of 3 different determinations made in triplicate. Error bars = 95% confidence intervals. (B) Various concentrations of acadesine were added to CD34+ cells (10^3^ cells/ml) from one CML patient growing in semisolid methylcellulose medium. After ten days in culture, MTT reagent (1 mg/ml) was added to the cell culture and the number of cell colonies was scored by the ImageJ quantification software.(C) 5×10^6^ K562 leukemic cell lines were implanted on both flanks in nude mice. After tumor establishment, animals received a daily intraperitoneal injection of either acadesine (50 mg/kg body weight in NaCl 0.9%). Results are expressed as the increase in tumor size as a function of time. The number of tumors was 12 and 14 for mice treated with control medium and acadesine containing medium respectively.

To assess the stability of acadesine we performed clonogenic outgrowth of K562 cells in methyl-cellulose (Figure 6A). Acadesine dose-dependently inhibited K562 colony formation at day 10. The growth inhibitory effect of acadesine was already detected at 0.25 mM and was maximal at 2.5 mM. The same experiments were performed with purified CD34+ from a CML patient. Acadesine at 0.5 mM was as potent as 1 µM Imatinib to reduce the number of colonies capable to grow in methyl-cellulose and no colony were detected at 1 mM acadesine (Figure 6B). Hence, the anti-leukemic effect of acadesine on K562 cells and CD34+ progenitor was long-standing and thus, we decided to test this compound on CML tumor formation in mice. To this end, we injected nude mice subcutaneously with 5.10^6^ K562 cells on both flanks. After one week, when tumors reached approximatively 250 mm^3^, mice were randomly separated in two groups. The first one received an intraperitoneal injection of NaCl 0.9% every day during 2 weeks whereas the remaining mice were treated with acadesine (50 mg/kg in NaCl 0.9%). Tumor size was evaluated at days 0, 7, 13, 16 and 20. Acadesine significantly reduced tumor formation in nude mice (Figure 6C). Statistic analysis of tumor size shows a robust reduction of 68% at day 16 and 51% at day 20, confirming the potent anti-leukemic effect of acadesine *in vivo*.

## Discussion

Acadesine has currently used in phaseI/II for the treatment of B-CLL [31]. In the present study we evaluated the potential anti-leukemic effect and the mechanism of action of acadesine on CML cell lines both *in vitro* and *in vivo*. The data presented herein demonstrate that acadesine exerts a potent anti-leukemic effect on different CML cell lines. Although acadesine does activate the AMPK pathway in CML cells, several lines of evidence indicate that this kinase is not involved in the anti-leukemic effect of acadesine. Firstly, AMPK knock-down by specific sh-RNA or si-RNA did not interfere with acadesine-mediated growth arrest and inhibition of cell metabolism. Secondly, the PKC inhibitors GFX and R0- efficiently inhibited the antileukemic effect of acadesine, but failed to affect AMPK phosphorylation in response to this AMPK activator (not shown). Importantly, acadesine activated several PKC isoforms, including α, β and γ in K562 cells. Altogether, our findings support the involvement of PKC but not AMPK in the anti-leukemic properties of acadesine. In several models of leukemia, acadesine has been reported to trigger AMPK activation, an effect accompanied by an important inhibition of their proliferation potential. This effect has often been associated with caspase activation and induction of apoptosis [32], [33]. Interestingly, this is clearly not the case in the present study, since we were unable to detect any increase in caspase activity upon acadesine stimulation of different CML cell lines. Accordingly, acadesine also failed to induce apoptosis in the different CML cell lines used in the present study. Thus, we conclude, that the anti-leukemic effect of acadesine did not involve induction of apoptosis in several CML cell lines.

We used a pharmacological approach to further decipher the signaling pathways by which acadesine exerts its anti-leukemic effect. Among the large panel of inhibitors tested, the anti-proliferative effect of acadesine was prevented only by the PKC inhibitor GFX. As GFX inhibited both conventional (c-PKC) and new PKC (n-PKC) isoforms, this finding strongly suggests that c and/or n-PKC mediate the anti-proliferative effect of acadesine on CML cell lines. We recently reported that K562 cells expressed several PKC isoforms [7] including conventional PKC α, β and γ and new PKC ε and θ Upon phorbol ester stimulation, these isoforms translocated from the soluble to the microsomal fraction with subsequent activation [7]. In the present study, we demonstrated for the first time that acadesine is a potent activator of the α, β, γ PKC isoforms and that the anti-proliferative effect of acadesine relies on PKC rather than AMPK activation. It has been recently reported that ischemic preconditioning activates the AMPK pathway in a PKCε-dependent manner suggesting a connection between the AMPK and PKC pathways [34]. It was proposed that PKCε may activate AMPK by phosphorylation but no evidence of this attractive possibility was presented in this study. Our results show that acadesine activated simultaneously and independently these two pathways in CML cell lines and that only PKC activation was involved in the anti-leukemic effect of acadesine. The exact mechanisms by which acadesine activates PKCs is currently unknown and further experiments are needed to decipher this novel and original signaling pathway.

It is well established that AMPK functions as an intracellular energy sensor regulating both metabolism and cell proliferation [35]. Under conditions of reduced nutrient avaibility, autophagy a process wherein catabolism of intracellular organelles generates energy allows cell survival. However, prolonged or massive autophagy can lead to non-apoptotic type II programmed cell death [36]. We observed that upon acadesine stimulation, K562 cells exhibited no evidence of apoptotic cell death, but finally died at 48 h from a non-apoptotic mode of cell death. It has been postulated that the dominant cell death phenotype is determined by the relative speed of the available cell death programs [37], [38]. Although several modes of death can be induced by the same effector only the most effective one, namely apoptosis is usually evident. In contrast to this model, acadesine promptly induced autophagy in CML cells leading to cell death in 24-48 h. We hypothesized that the death induced by acadesine in CML cells may represent type II programmed cell death. Confocal and electron microscopy analysis together with measurement of LC3 accumulation confirmed that acadesine was a potent inducer of autophagic cell death in different CML cell lines. There are compelling evidence that acadesine may act as an anti-proliferative agent in epithelial cancerous cells [32], [39], [40] and in some leukemic cell lines [41], [42]. Regarding leukemia, acadesine activates and induces apoptosis in B-CLL [42] and is cytotoxic for childhood acute lymphoblastic leukemia [41]. In both cases, acadesine was found to increase AMPK activity but it is not known from these studies whether or not activation of AMPK is responsible for the observed anti-proliferative effects. As AMPK is not involved in the anti-leukemic effect of acadesine in CML cells, it would be interesting to determine if it is also the case in other leukemia.

Our data established also that treatment of mice with engrafted CML tumors with acadesine, mediated tumor regression. Such an anti-tumoral effect of acadesine has already been reported on the growth of prostatic tumor in nude mice [43], but to our knowledge our data are the first to demonstrate that acadesine might induce tumor regression of leukemic cells *in vivo*. Importantly, our data also showed that acadesine is very effective in Imatinib-resistant K562 cells and Ba/F3-T315I cells killing and CD34+ cells from CML patient.

Finally, based on our findings we propose an original model in which the anti-leukemic effect of acadesine is dependent on PKC-mediated induction of autophagic cell death but independent of AMPK activation and apoptosis. The relation between PKC activation and autophagy is not clear, but PMA through PKCα activation has been shown to increase the volume of autophagosomes in hepatocytes [44]. In the same line, we have shown recently that the phorbol ester and PKC activator PMA promotes autophagy in K562 cells [45]. This is consistent with the findings reported herein regarding the effect of acadesine on autophagy.

To the best of our knowledge, our work provides the first evidence that PKC-mediated induction of autophagy is required for the anti-leukemic effect of acadesine. Therefore, in addition to its effect in B-CLL, acadesine might find therapeutical application in Imatinib-resistant patients.

### Ethical Statement

This study was conducted according to the principles expressed in the Declaration of Helsinki. Animal studies were approved by the Institutional Animal Care and Use Committee of the Centre Mediterraneen de Medecine Moleculaire (INSERM U895), Agreement 0752.

## Supporting Information

Figure S1 Acadesine Induces loss of cell viability in different CML cell lines in an apoptotic independent manner. JURL-MK1 cells (A) were incubated for 48 h at 37°C with 1 mM acadesine or 1 µM Imatinib. Cell metabolism was measured by the XTT assay as described in Figure 1A. K562 parental and Ima-R cells (B) LAMA-84 cells (C) and Ba/F3-WT and T315I cells (D) were incubated with increasing doses of acadesine (0.1-2 mM). Cell metabolism was measured by the XTT assay as described above. Error bars = 95% confidence intervals. K562 (E) and Ima-R K562 cells (F) were incubated for 48 h with either 1 µM Imatinib or acadesine (0.5 and 1 mM). Detection of apoptotic cells was determined using annexinV/PI staining and FACS analysis.
(2.05 MB TIF)

Figure S2 Acadesine induces significant changes in cell shape and morphology in K562 cells. (A) K562 cells were incubated for 48 h at 37°C in the presence or the absence of 1 mM acadesine. Then, cells were cytospun on a slide, air-dried and stained with May-Grünwald Giemsa. Slides were observed with an inverted microscope at different magnifications. (B) K562 cells were incubated for different times at 37°C in the presence or the absence of 1 mM acadesine and treated as described in (A).
(5.90 MB TIF)

Figure S3 Acadesine triggers autophagy and p62/SQSTM1 accumulation in CML cells from different origins. K562 and Ima-R K562 cells (A), Lama-84 cells (B) and BaF/3 WT and T315I cells (C) were incubated for 48 h with 1 mM acadesine. Proteins were extracted and analyzed by western blotting using phosphoS6RP, S6RP, LC3 and p62/SQSTM1 antibodies. Actin was used as a loading control.
(3.30 MB TIF)

Figure S4 GFX induces inhibition of vacuole formation in acadesine treated CML cells. Electron microscopy images showing ultrastructural features of: (A) and (B) untreated K562 cells. (C) and (D) K562 cells treated with 5 µM GFX (E), (F) and (I) K562 cells treated with 1 mM acadesine (G) and (H) K562 cells treated with the combination of acadesine (1 mM) and GFX (5 µM).
(5.02 MB TIF)

## References

[1] Groffen J, Stephenson JR, Heisterkamp N, de Klein A, Bartram CR (1984). Philadelphia chromosomal breakpoints are clustered within a limited region, bcr, on chromosome 22.. Cell.

[2] Ren R (2002). The molecular mechanism of chronic myelogenous leukemia and its therapeutic implications: studies in a murine model.. Oncogene.

[3] Koschmieder S, Gottgens B, Zhang P, Iwasaki-Arai J, Akashi K (2005). Inducible chronic phase of myeloid leukemia with expansion of hematopoietic stem cells in a transgenic model of BCR-ABL leukemogenesis.. Blood.

[4] Neering SJ, Bushnell T, Sozer S, Ashton J, Rossi RM (2007). Leukemia stem cells in a genetically defined murine model of blast-crisis CML.. Blood.

[5] Deininger MW, Goldman JM, Melo JV (2000). The molecular biology of chronic myeloid leukemia.. Blood.

[6] Jacquel A, Herrant M, Legros L, Belhacene N, Luciano F (2003). Imatinib induces mitochondria-dependent apoptosis of the Bcr-Abl-positive K562 cell line and its differentiation toward the erythroid lineage.. Faseb J.

[7] Jacquel A, Herrant M, Defamie V, Belhacene N, Colosetti P (2006). A survey of the signaling pathways involved in megakaryocytic differentiation of the human K562 leukemia cell line by molecular and c-DNA array analysis.. Oncogene.

[8] Steelman LS, Pohnert SC, Shelton JG, Franklin RA, Bertrand FE (2004). JAK/STAT, Raf/MEK/ERK, PI3K/Akt and BCR-ABL in cell cycle progression and leukemogenesis.. Leukemia.

[9] Buchdunger E, Zimmermann J, Mett H, Meyer T, Muller M (1995). Selective inhibition of the platelet-derived growth factor signal transduction pathway by a protein-tyrosine kinase inhibitor of the 2-phenylaminopyrimidine class.. Proc Natl Acad Sci U S A.

[10] Heinrich MC, Griffith DJ, Druker BJ, Wait CL, Ott KA (2000). Inhibition of c-kit receptor tyrosine kinase activity by STI 571, a selective tyrosine kinase inhibitor.. Blood.

[11] Dan S, Naito M, Tsuruo T (1998). Selective induction of apoptosis in Philadelphia chromosome-positive chronic myelogenous leukemia cells by an inhibitor of BCR - ABL tyrosine kinase, CGP 57148.. Cell Death Differ.

[12] Fang G, Kim CN, Perkins CL, Ramadevi N, Winton E (2000). CGP57148B (STI-571) induces differentiation and apoptosis and sensitizes Bcr-Abl-positive human leukemia cells to apoptosis due to antileukemic drugs.. Blood.

[13] Druker BJ, Sawyers CL, Kantarjian H, Resta DJ, Reese SF (2001). Activity of a specific inhibitor of the BCR-ABL tyrosine kinase in the blast crisis of chronic myeloid leukemia and acute lymphoblastic leukemia with the Philadelphia chromosome.. N Engl J Med.

[14] Kahn BB, Alquier T, Carling D, Hardie DG (2005). AMP-activated protein kinase: ancient energy gauge provides clues to modern understanding of metabolism.. Cell Metab.

[15] Long YC, Zierath JR (2006). AMP-activated protein kinase signaling in metabolic regulation.. J Clin Invest.

[16] Jacobs RL, Lingrell S, Dyck JR, Vance DE (2007). Inhibition of hepatic phosphatidylcholine synthesis by 5-aminoimidazole-4-carboxamide-1-beta-4-ribofuranoside is independent of AMP-activated protein kinase activation.. J Biol Chem.

[17] Kuo CL, Ho FM, Chang MY, Prakash E, Lin WW (2008). Inhibition of lipopolysaccharide-induced inducible nitric oxide synthase and cyclooxygenase-2 gene expression by 5-aminoimidazole-4-carboxamide riboside is independent of AMP-activated protein kinase.. J Cell Biochem.

[18] Guigas B, Bertrand L, Taleux N, Foretz M, Wiernsperger N (2006). 5-Aminoimidazole-4-carboxamide-1-beta-D-ribofuranoside and metformin inhibit hepatic glucose phosphorylation by an AMP-activated protein kinase-independent effect on glucokinase translocation.. Diabetes.

[19] Puissant A, Grosso S, Jacquel A, Belhacene N, Colosetti P (2008). Imatinib mesylate-resistant human chronic myelogenous leukemia cell lines exhibit high sensitivity to the phytoalexin resveratrol.. Faseb J.

[20] Herrant M, Luciano F, Loubat A, Auberger P (2002). The protective effect of phorbol esters on Fas-mediated apoptosis in T cells. Transcriptional and postranscriptional regulation.. Oncogene.

[21] Herrant M, Jacquel A, Marchetti S, Belhacene N, Colosetti P (2004). Cleavage of Mcl-1 by caspases impaired its ability to counteract Bim-induced apoptosis.. Oncogene.

[22] Jacquel A, Colosetti P, Grosso S, Belhacene N, Puissant A (2007). Apoptosis and erythroid differentiation triggered by Bcr-Abl inhibitors in CML cell lines are fully distinguishable processes that exhibit different sensitivity to caspase inhibition.. Oncogene.

[23] Bellodi C, Lidonnici MR, Hamilton A, Helgason GV, Soliera AR (2009). Targeting autophagy potentiates tyrosine kinase inhibitor-induced cell death in Philadelphia chromosome-positive cells, including primary CML stem cells.. J Clin Invest.

[24] Klionsky DJ, Abeliovich H, Agostinis P, Agrawal DK, Aliev G (2008). Guidelines for the use and interpretation of assays for monitoring autophagy in higher eukaryotes.. Autophagy.

[25] Conus S, Simon HU (2008). Cathepsins: key modulators of cell death and inflammatory responses.. Biochem Pharmacol.

[26] Ben Sahra I, Laurent K, Loubat A, Giorgetti-Peraldi S, Colosetti P (2008). The antidiabetic drug metformin exerts an antitumoral effect in vitro and in vivo through a decrease of cyclin D1 level.. Oncogene.

[27] Inoki K, Zhu T, Guan KL (2003). TSC2 mediates cellular energy response to control cell growth and survival.. Cell.

[28] Schaefer M, Albrecht N, Hofmann T, Gudermann T, Schultz G (2001). Diffusion-limited translocation mechanism of protein kinase C isotypes.. Faseb J.

[29] Tanimura A, Nezu A, Morita T, Hashimoto N, Tojyo Y (2002). Interplay between calcium, diacylglycerol, and phosphorylation in the spatial and temporal regulation of PKCalpha-GFP.. J Biol Chem.

[30] Mari B, Guerin S, Maulon L, Belhacene N, Farahi Far D (1997). Endopeptidase 24.11 (CD10/NEP) is required for phorbol ester-induced growth arrest in Jurkat T cells.. Faseb J.

[31] (2008). Acadesine: AICA riboside, ARA 100, arasine, GP 1 110.. Drugs R D.

[32] Rattan R, Giri S, Singh AK, Singh I (2005). 5-Aminoimidazole-4-carboxamide-1-beta-D-ribofuranoside inhibits cancer cell proliferation in vitro and in vivo via AMP-activated protein kinase.. J Biol Chem.

[33] Campas C, Santidrian AF, Domingo A, Gil J (2005). Acadesine induces apoptosis in B cells from mantle cell lymphoma and splenic marginal zone lymphoma.. Leukemia.

[34] Nishino Y, Miura T, Miki T, Sakamoto J, Nakamura Y (2004). Ischemic preconditioning activates AMPK in a PKC-dependent manner and induces GLUT4 up-regulation in the late phase of cardioprotection.. Cardiovasc Res.

[35] Motoshima H, Goldstein BJ, Igata M, Araki E (2006). AMPK and cell proliferation–AMPK as a therapeutic target for atherosclerosis and cancer.. J Physiol.

[36] Galluzzi L, Vicencio JM, Kepp O, Tasdemir E, Maiuri MC (2008). To die or not to die: that is the autophagic question.. Curr Mol Med.

[37] Bursch W (2001). The autophagosomal-lysosomal compartment in programmed cell death.. Cell Death Differ.

[38] Broker LE, Kruyt FA, Giaccone G (2005). Cell death independent of caspases: a review.. Clin Cancer Res.

[39] Su RY, Chao Y, Chen TY, Huang DY, Lin WW (2007). 5-Aminoimidazole-4-carboxamide riboside sensitizes TRAIL- and TNF{alpha}-induced cytotoxicity in colon cancer cells through AMP-activated protein kinase signaling.. Mol Cancer Ther.

[40] Kim YM, Hwang JT, Kwak DW, Lee YK, Park OJ (2007). Involvement of AMPK signaling cascade in capsaicin-induced apoptosis of HT-29 colon cancer cells.. Ann N Y Acad Sci.

[41] Sengupta TK, Leclerc GM, Hsieh-Kinser TT, Leclerc GJ, Singh I (2007). Cytotoxic effect of 5-aminoimidazole-4-carboxamide-1-beta-4-ribofuranoside (AICAR) on childhood acute lymphoblastic leukemia (ALL) cells: implication for targeted therapy.. Mol Cancer.

[42] Campas C, Lopez JM, Santidrian AF, Barragan M, Bellosillo B (2003). Acadesine activates AMPK and induces apoptosis in B-cell chronic lymphocytic leukemia cells but not in T lymphocytes.. Blood.

[43] Swinnen JV, Beckers A, Brusselmans K, Organe S, Segers J (2005). Mimicry of a cellular low energy status blocks tumor cell anabolism and suppresses the malignant phenotype.. Cancer Res.

[44] Larocca MC, Ochoa EJ, Rodriguez Garay EA, Marinelli RA (2002). Protein kinase C-dependent inhibition of the lysosomal degradation of endocytosed proteins in rat hepatocytes.. Cell Signal.

[45] Colosetti P, Puissant A, Robert G, Luciano F, Jacquel A (2009). Autophagy is an important event for megakaryocytic differentiation of the chronic myelogenous leukemia K562 cell line.. Autophagy.

