# Supplementary figures and images for: Acadesine Kills Chronic Myelogenous Leukemia (CML) Cells through PKC-Dependent Induction of Autophagic Cell Death
Source: PLoS One. 2009 Nov 18;4(11):e7889. doi: 10.1371/journal.pone.0007889 (PMC2775681; doi:10.1371/journal.pone.0007889)

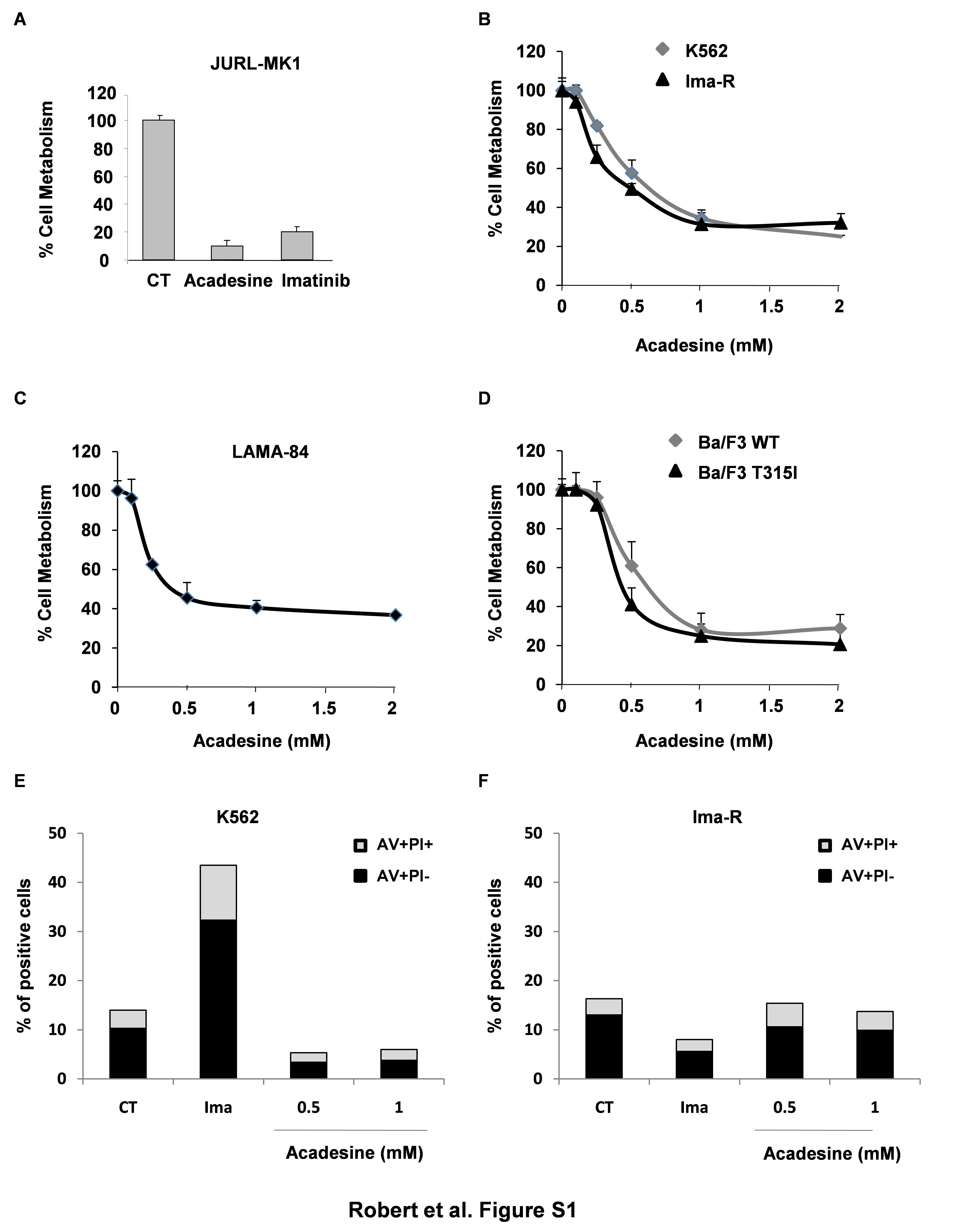

Supplement: Figure S1 — Acadesine Induces loss of cell viability in different CML cell lines in an apoptotic independent manner. JURL-MK1 cells (A) were incubated for 48 h at 37°C with 1 mM acadesine or 1 µM Imatinib. Cell metabolism was measured by the XTT assay as described in Figure 1A. K562 parental and Ima-R cells (B) LAMA-84 cells (C) and Ba/F3-WT and T315I cells (D) were incubated with increasing doses of acadesine (0.1-2 mM). Cell metabolism was measured by the XTT assay as described above. Error bars = 95% confidence intervals. K562 (E) and Ima-R K562 cells (F) were incubated for 48 h with either 1 µM Imatinib or acadesine (0.5 and 1 mM). Detection of apoptotic cells was determined using annexinV/PI staining and FACS analysis. (2.05 MB TIF) [file pone.0007889.s001.tif]

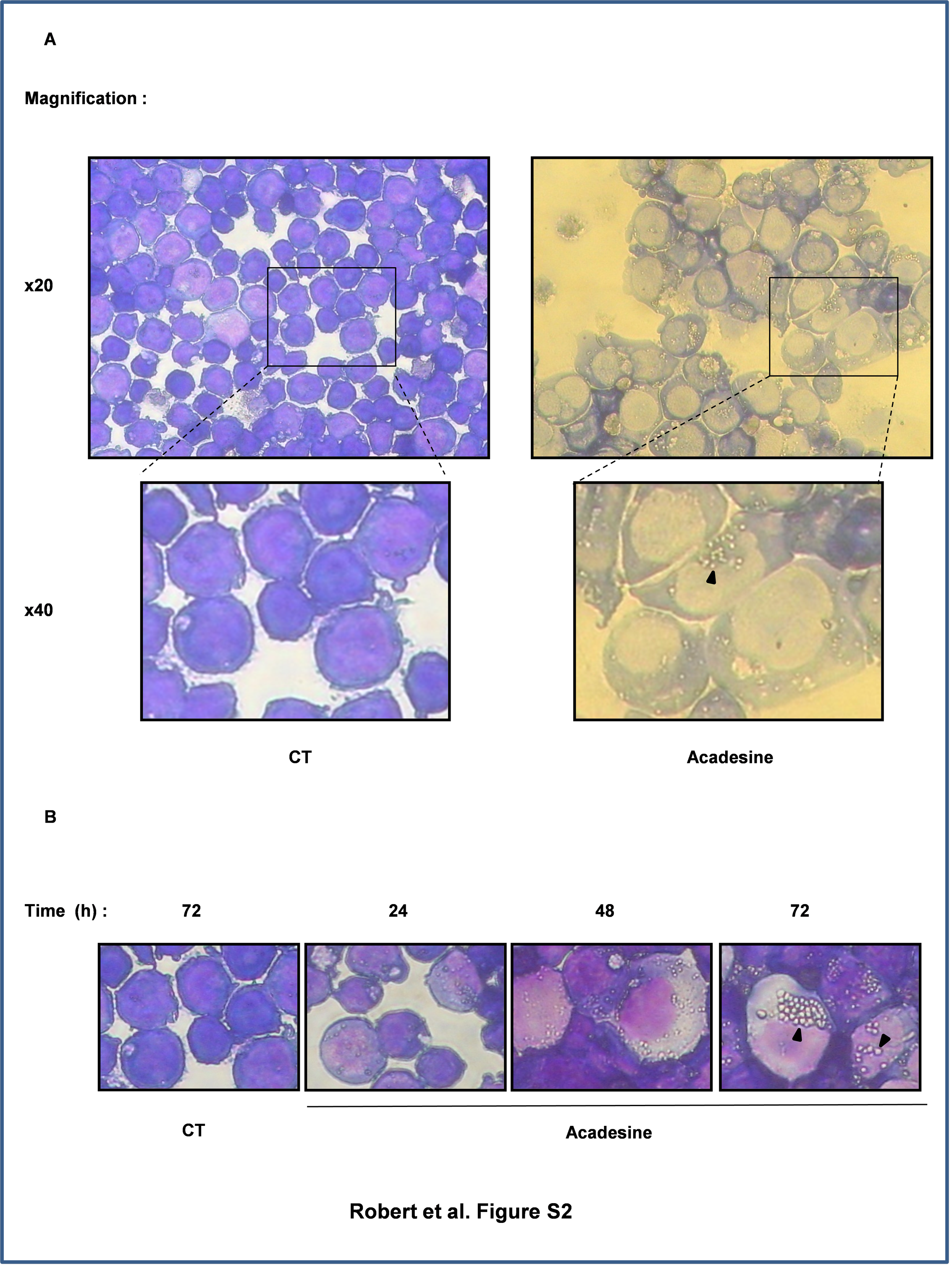

Supplement: Figure S2 — Acadesine induces significant changes in cell shape and morphology in K562 cells. (A) K562 cells were incubated for 48 h at 37°C in the presence or the absence of 1 mM acadesine. Then, cells were cytospun on a slide, air-dried and stained with May-Grünwald Giemsa. Slides were observed with an inverted microscope at different magnifications. (B) K562 cells were incubated for different times at 37°C in the presence or the absence of 1 mM acadesine and treated as described in (A). (5.90 MB TIF) [file pone.0007889.s002.tif]

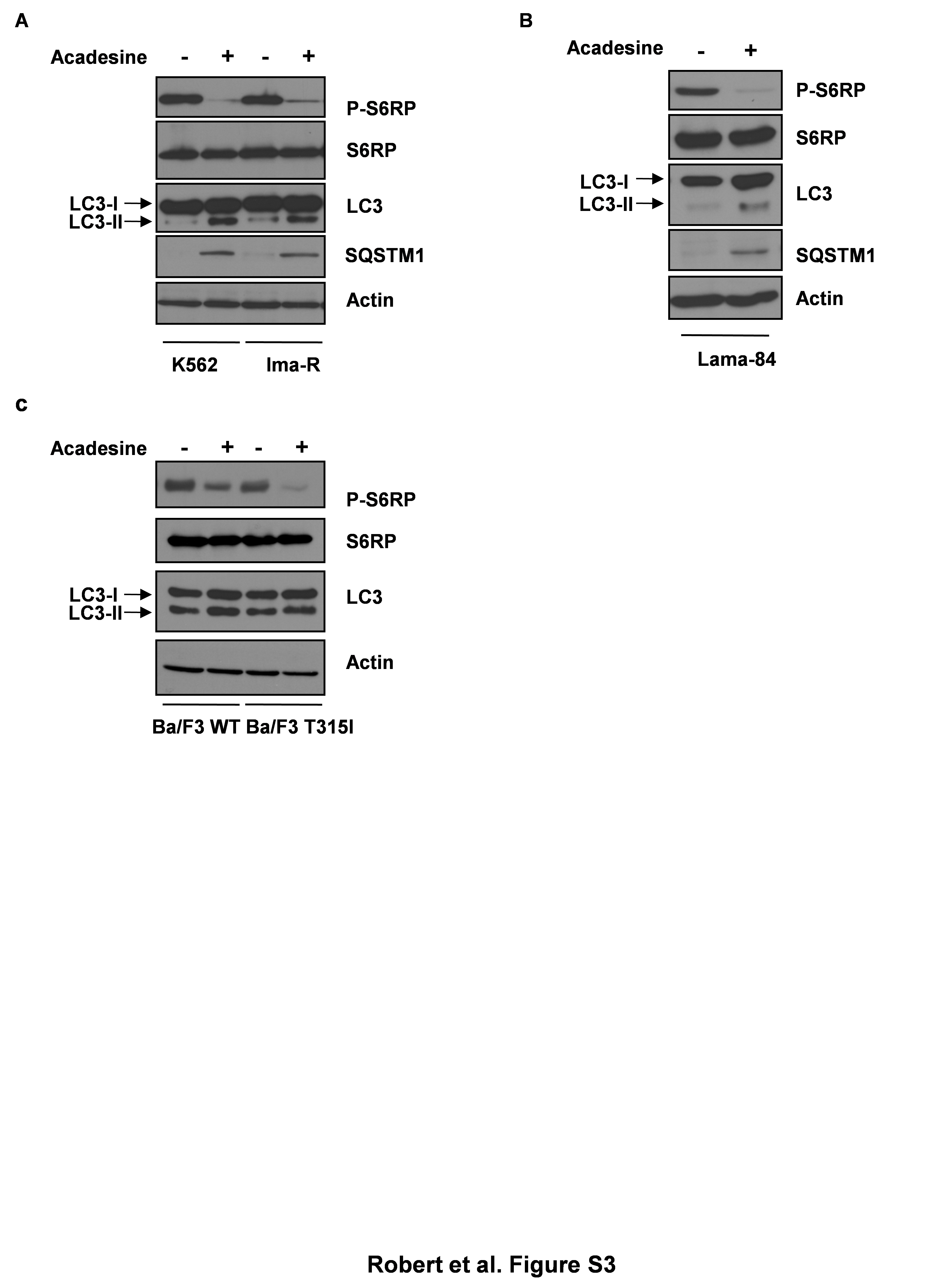

Supplement: Figure S3 — Acadesine triggers autophagy and p62/SQSTM1 accumulation in CML cells from different origins. K562 and Ima-R K562 cells (A), Lama-84 cells (B) and BaF/3 WT and T315I cells (C) were incubated for 48 h with 1 mM acadesine. Proteins were extracted and analyzed by western blotting using phosphoS6RP, S6RP, LC3 and p62/SQSTM1 antibodies. Actin was used as a loading control. (3.30 MB TIF) [file pone.0007889.s003.tif]

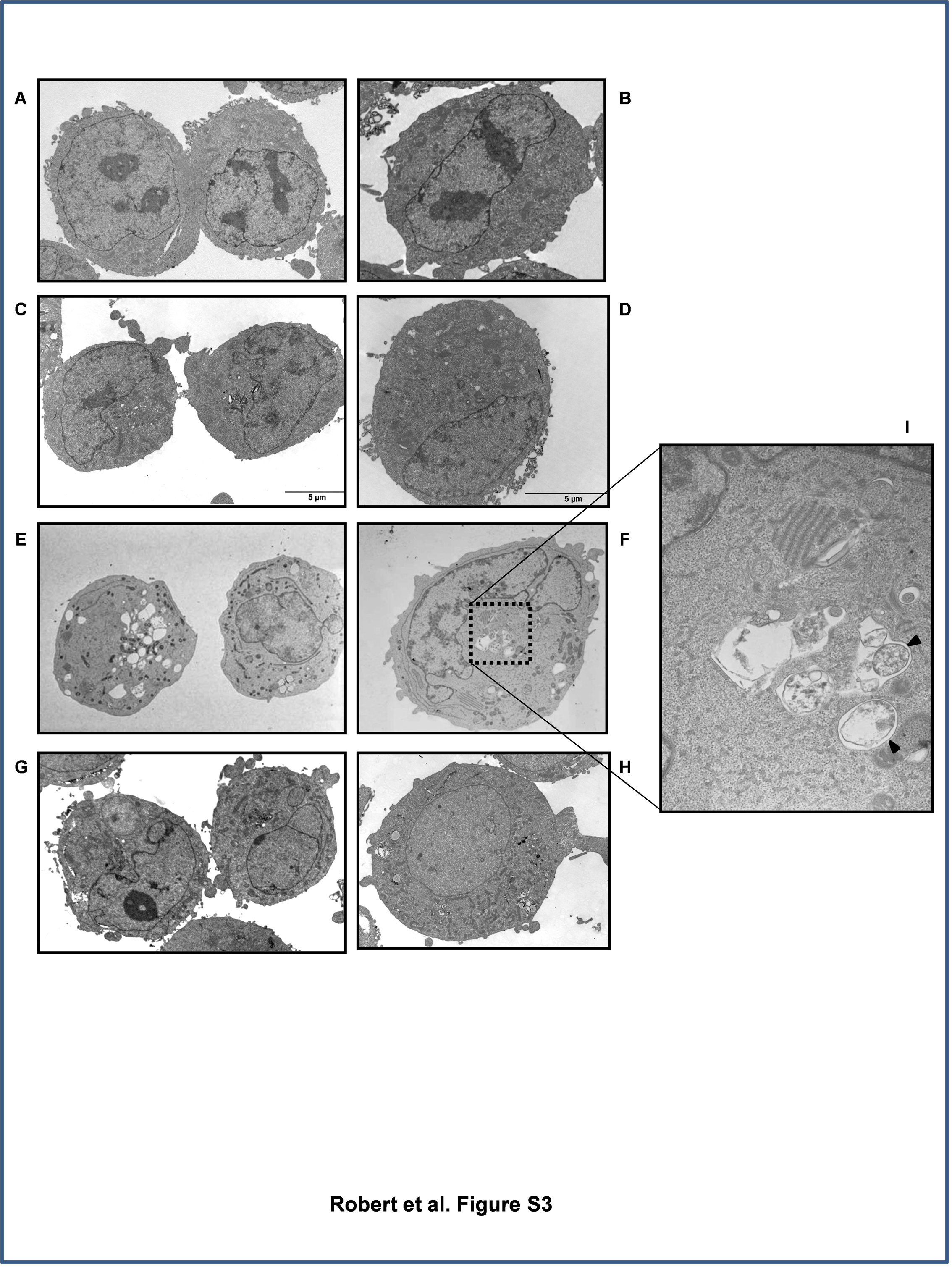

Supplement: Figure S4 — GFX induces inhibition of vacuole formation in acadesine treated CML cells. Electron microscopy images showing ultrastructural features of: (A) and (B) untreated K562 cells. (C) and (D) K562 cells treated with 5 µM GFX (E), (F) and (I) K562 cells treated with 1 mM acadesine (G) and (H) K562 cells treated with the combination of acadesine (1 mM) and GFX (5 µM). (5.02 MB TIF) [file pone.0007889.s004.tif]
